# A case–control study comparing the incidence of early symptoms in pancreatic and biliary tract cancer

**DOI:** 10.1136/bmjopen-2014-005720

**Published:** 2014-11-19

**Authors:** M G Keane, L Horsfall, G Rait, S P Pereira

**Affiliations:** 1 Institute for Liver and Digestive Health, University College London, London, UK; 2 Research Department of Primary Care and Population Health, University College London, London, UK

**Keywords:** PRIMARY CARE

## Abstract

**Objectives:**

Pancreatic ductal adenocarcinoma (PDAC) and biliary tract cancers (BTC) are often diagnosed late and at an advanced stage. Population-based screening programmes do not exist and diagnosis is primarily dependent on symptom recognition. Recently symptom-based cancer decision support tools (CDSTs) have been introduced into primary care practices throughout the UK to support general practitioners (GPs) in identifying patients with suspected PDAC. However, future refinement of these tools to improve their diagnostic accuracy is likely to be necessary.

**Setting:**

The Health Improvement Network (THIN) is a primary care database, which includes more than 11 million electronic patient records, from 562 GP practices in the UK.

**Participants:**

All patients with a diagnosis of PDAC or BTC between 2000 and 2010 were included in the study along with six matched controls; 2773 patients with PDAC, 848 patients with BTC and 15 395 controls.

**Primary and secondary outcome measures:**

The primary aim of this study was to determine the early symptom profiles of PDAC and BTC. Secondary aims included comparing early symptom trends between BTC and PDAC, defining symptom onset in PDAC and evaluating trends in routine blood tests nearest to the time of diagnosis.

**Results:**

In the year prior to diagnosis, patients with PDAC visited their GP on a median of 18 (IQR 11–27) occasions. PDAC was associated with 11 alarm symptoms and BTC with 8. Back pain (OR 1.33 (95% CI 1.18 to 1.49) p<0.001), lethargy (1.42 (95% CI 1.25 to 1.62) p<0.001) and new onset diabetes (OR 2.46 (95% CI 2.16 to 2.80)) were identified as unique features of PDAC.

**Conclusions:**

PDAC and BTC are associated with numerous early alarm symptoms. CDSTs are therefore likely to be useful in identifying these tumours at an early stage. Inclusion of unique symptoms, symptoms with an early onset and routinely performed blood tests is likely to further improve the sensitivity of these tools.

**Strengths and limitations of this study**

- A significant strength of this study is that the Health Improvement Network (THIN) database includes routinely recorded primary care data that is not subject to recall bias.
- The database includes a large cohort of patients with pancreatic ductal adenocarcinoma and biliary tract cancers (BTC).
- The patient population within the database has been shown to be representative of the UK population.
- This is the first study to evaluate early symptoms of BTC in primary care.
- A patient's final diagnosis within the THIN database is dependent on correct coding by their general practitioner. However, this has generally been found to be accurate in patients with cancer, when electronic records have been compared to hospital correspondence or written medical records.
- The study was limited to Read code analysis alone. A potential concern is that additional information is contained in the ‘free-text’ area of the notes. Previous studies that have explored this and have found it to only occur rarely.
- Currently, information about histology, stage of disease at diagnosis or treatment received is not linked to presenting symptoms within the THIN database. It is therefore impossible to determine which symptoms are associated with the earliest stages of the disease and would therefore offer the greatest opportunity for early intervention and treatment. However, linkage of THIN data to Hospital Episode Statistics (HES) data is currently underway.
- Although conditional logistic regression was not utilised to take advantage of our matched data, the study's sample size was large enough to make losses in statistical power negligible.
- The primary aim of the figures in this study was to present a visual representation of general trends within the data.

## Background

Pancreatic ductal adenocarcinoma (PDAC) and biliary tract cancers (BTC) are lethal tumours that are often diagnosed late when the disease is at an advanced stage and no longer amenable to curative surgical resection.^1^ ^2^ PDAC is the ninth most common cancer in the UK with more than 8000 cases diagnosed each year compared with less than 2000 cases of BTC. Overall 5-year survival in both tumours is less than 4%.^2^ ^3^ Despite advances in diagnostic technology and the identification of a number of promising biomarkers, impact on survival has been limited and novel diagnostic strategies are therefore urgently needed.^4^

Recently prediagnostic symptom profiles have been investigated as a method of enabling earlier diagnosis, in a number of common cancers including PDAC.^5^ ^6–8^ The diagnosis of PDAC is heralded by the insidious onset of a heterogeneous collection of symptoms. Although symptom profiles are recognised to vary between patients with PDAC, certain symptoms appear to occur with sufficient frequency to be useful as early diagnostic markers of the disease. To date, prediagnostic symptom profiles for PDAC have largely been defined through postdiagnosis retrospective interview studies of secondary care patients^8^ and through the interrogation of large primary care databases with predefined symptom lists.^6^ ^7^ Almost no studies have explored the symptom profile of BTC.

Defining the early symptom profiles of PDAC has enabled the development of symptom-based cancer decision support tools (CDSTs). Recently these tools have been introduced into primary care practices across 15 cancer networks, throughout the UK.^6^ Their impact on referral practice is subject to an ongoing audit.^9^ CDSTs for PDAC have been validated independently within other primary care data sets. Initial results suggest that although they can effectively discriminate patients with PDAC, they may overestimate cancer risk in certain groups, in particular in older patients.^10^ Future modification of existing tools to improve their overall diagnostic accuracy is therefore likely to be required.

Patients with PDAC frequently encounter a number of delays during their route to diagnosis.^11^ Although PDAC is no longer considered to be a symptomatically silent disease, debate exists about how long patients are symptomatic for and if symptoms occur simultaneously or sequentially. A recent qualitative interview study suggested very early symptoms might actually be intermittent and therefore reassuring to patients leading them to ignore them until they increase in severity or other symptoms arise.^12^ Once symptomatic, large primary care database studies and patient surveys indicate that patients with PDAC visit their general practitioner (GP) frequently with alarm symptoms in the months and years prior to diagnosis.^6^ ^7^ ^11^ ^13^ However, almost half of patients are still diagnosed as a result of an emergency presentation to hospital.^11^ Reasons why the disease is not identified earlier are complex. The average GP will only see one new case of PDAC every 5 years and alarm symptoms overlap with a number of other more common benign and malignant conditions, as a result it is recognised as a very challenging disease to identify at an early stage.^11^ Simple screening tests do not exist and very few GPs have access to cross-sectional imaging; therefore, diagnosis of PDAC in primary care is primarily dependent on symptom recognition. How alarm symptoms for PDAC overlap with other conditions has rarely been evaluated, and it is unclear if there are certain symptoms or combinations of symptoms that are unique to PDAC. In addition, very little is known about what features of the disease prompt GPs to suspect cancer and initiate investigations and referrals. Current National Institute for Health and Care Excellence (NICE) referral guidelines for suspected cancer in primary care contain limited specific information on the best method for referring patients with suspected PDAC or BTC for further investigation, but new guidelines are underway.^14^

The primary aim of this study was therefore to determine the early symptom profiles of PDAC and BTC in a large primary care cohort. Secondary aims include comparing early symptom trends between BTC and PDAC, defining symptom onset in PDAC and evaluating trends in routine blood tests nearest to the time of diagnosis.

## Methods

### Data source

In the UK most GPs record patient data electronically. A subset of GP practices have opted to provide anonymous electronic patient records for use in clinical and epidemiological research. The Health Improvement Network (THIN) is a primary care database, which includes more than 11 million electronic patient records, from 562 GP practices, covering around 6% of the UK population (http://csdmruk.cegedim.com/). The data are broadly representative of the UK general practice population in terms of demographics and consultation behaviour.^15^ ^16^ Diagnoses, symptoms and referrals to secondary care are electronically recorded using the Read code system.^17^ Clinical diagnoses recorded by GPs electronically have recently been shown to be accurate compared with other reliable sources.^16^ ^18^ All drug prescriptions and variables such as body mass index (BMI), blood pressure, smoking status, alcohol intake and laboratory results are also recorded.

### Study design

A case–control design was used to compare ‘alarm’ symptoms and commonly performed blood test results in patients with a diagnosis of PDAC or BTC. Unaffected controls were matched for age, sex, practice and year of diagnosis.

### Study population

**Figure 1:**
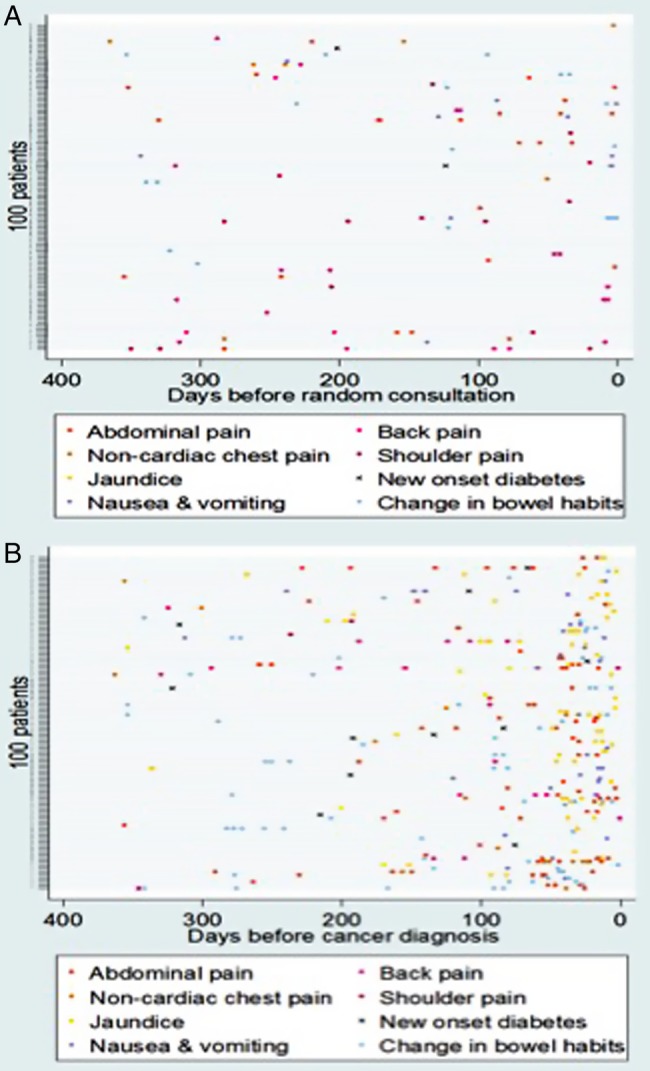
(A) Incidence and timing of common presenting symptoms in 100 matched controls 1 year before a random consultation. (B) Incidence and timing of common presenting symptoms in 100 randomly selected patients with pancreatic cancer in the year prior to diagnosis.

All patients with a Read code diagnosis of PDAC or BTC between 1 January 2000 and 31 December 2010 were extracted from the database. Read code lists to identify diagnosed patients were developed using previously described methodology.^19^ The date of diagnosis was set as the index date and for control patients a random consultation date was selected to become the index date. All patients were required to have contributed 2 years of data prior to the index date. Two years was selected as the time period of interest based on preliminary data suggesting alarm symptoms were uncommon beyond this time period (figure 1A, B). To help ensure the data analysed were of adequate quality, only patients from GP practices which had achieved both acceptable mortality recording^20^ and an acceptable computer usage^21^ were included.

The control sample contained randomly selected patients without a diagnosis of PDAC or BTC. Stratified sampling within the same GP practices from where patients with a cancer diagnosis were identified was used to ensure control patients had similar characteristics to those with a cancer diagnosis in terms of age, sex, practice and equivalent year of consultation (control group) to year of diagnosis (cancer group). Up to six control patients were selected per patient with a cancer diagnosis.

### Outcomes

Alarm symptoms and laboratory tests were selected based on clinical knowledge and the existing literature.^6^ ^7^ ^22–31^ To ensure that no symptoms had been missed by the literature review, Read codes for 10% of patients with PDAC (n=296) were reviewed in their entirety to identify any additional common or biologically plausible symptoms (table 1). For each individual symptom, frequency, median onset and average number of presentations were recorded. Symptoms were grouped according to pathological aetiology and onset (greater or less than 6 months prior to diagnosis). All symptoms with a frequency of greater than 5% were identified as potential alarm symptoms and included in the subsequent case–control study (table 1).

**Table 1:** Frequency and onset of common and biologically plausible symptoms in a 10% cohort of patients with pancreatic ductal adenocarcinoma

Symptom*	N=296 (%)	Median symptom onset—days prior to diagnosis [range]	Mean number of presentations with symptom prior to diagnosis [range]
**Pain**			
Abdominal pain	130 (44)	106 [1–1092]	1.20 [0–12]
Back pain	90 (30)	483 [7–1092]	0.54 [0–6]
Non cardiac chest pain	39 (13)	159 [10–1093]	0.18 [0–5]
Shoulder pain	21 (7)	671 [48–1095]	0.13 [0–5]
**Upper GI symptoms**			
Dyspepsia/reflux	77 (26)	136 [12–1095]	0.52 [0–15]
Nausea and vomiting	58 (20)	73 [1–1073]	0.30 [0–13]
Abdominal mass	11 (4)	49 [0–255]	0.04 [0–1]
Bloating	9 (3)	87 [27–607]	0.00 [0–1]
Upper gastrointestinal bleeding	8 (3)	49 [2–689]	0.00 [0–1]
Dysphagia	7 (2)	227 [0–603]	0.03 [0–1]
Hepatomegaly	3 (1)	10 [0–68]	0.01 [0–1]
**Bile duct obstruction**			
Jaundice	104 (35)	31 [0–648]	0.35 [0–1]
Pruritus	23 (8)	114 [13–1059]	0.10 [0–5]
**Lower GI symptoms**			
Change in bowel habit	104 (35)	188 [0–1078]	0.73 [0–14]
**Pancreatic dysfunction**			
Pancreatitis	11 (4)	108 [21–922]	0.03 [0–1]
Steattorhoea	4 (1)	62 [0–593]	0.02 [0–1]
**Other constitutional symptoms**			
Weight loss	29 (10)	144 [14–937]	0.09 [0–1]
Lethargy	23 (8)	219 [8–988]	0.10 [0–3]
Anorexia	14 (5)	46 [2–337]	0.00 [0–1]
DVT/PE	11 (4)	24 [0–1084]	0.04 [0–1]
Insomnia	6 (2)	45 [34–74]	0.00 [0–1]
Fracture	4 (1)	78 [0–242]	0.00 [0–1]
Change in taste/smell	2 (0.7)	40 [38–42]	0.00 [0–1]

*Each symptom was assessed independently.

[▪]=Symptom onset >6 months prior to diagnosis. [ ]=Symptom onset <6 months prior to diagnosis.

GI, gastrointestinal.

Laboratory tests were restricted to routinely performed tests to ensure adequate numbers were recorded for the control population and included haemoglobin and liver function tests: serum bilirubin, alkaline phosphatase (ALP), alanine aminotransferase (ALT).

### Covariates

Age, gender, time period and Townsend score, smoking status and BMI were selected as potential confounders. Where multiple measures of BMI and smoking status were recorded, the earliest record in the 2-year time frame from the index date was selected. Deprivation was examined using quintiles of Townsend score from ‘one’ (least deprived) to ‘five’ (most deprived). The Townsend score is a combined measure of owner occupation, car ownership, overcrowding and unemployment based on a patient's postcode and linkage to population census data for 2001 for approximately 150 households in that postal area.

### Statistical analyses

Multivariable logistic regression was used to estimate the ORs for symptoms in the 2 years prior to PDAC or BTC diagnosis versus the 2 years prior to the index date in patients with and without cancer. Linear regression was used to estimate adjusted mean differences in clinical measures between patients with and without cancer. Although many of these laboratory values are slightly skewed, we decided to analyse the data without transformation—the large sample size means the statistical analyses should be robust to deviation from normality and transformation can make results difficult to interpret. To account for data clustering within GP practice, we used a multilevel regression model with the practice identifier entered as a random effect. All p values were two-tailed and a value of less than 5% (≤0.05) was considered statistically significant. All analyses were done using Stata V.12.1.

## Results

### Read code analysis of a subgroup 296 patients with PDAC

**Figure 2:**
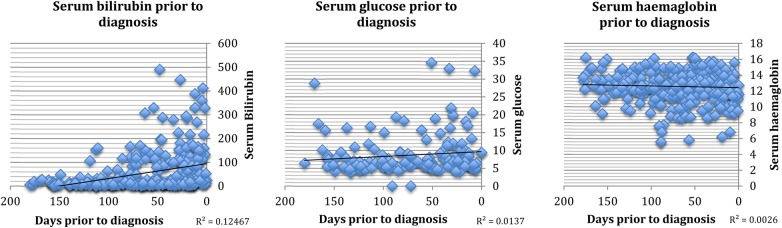
Trends in commonly performed blood tests in the year prior to diagnosis (bilirubin N=345 blood tests from 192 patients, glucose N=188 blood tests from 118 patients, haemoglobin N=335 blood tests from 195 patients).

The Read codes of 10% of randomly selected patients with PDAC (296 cases) were reviewed in their entirety. In this group, symptoms were common (table 1); 91% (268/296) had relevant symptoms in the 2 years prior to diagnosis. Patients attended their GP on a median of 3 occasions with alarm symptoms (range 0–22) during this period but visits did cluster nearest to the time of diagnosis (figure 1A, B). In those who were symptomatic, 51% (136/268) reattended with the same symptom during the 2-year time period and 75% (202/268) reattended with an alternative alarm symptom. Common alarm symptoms that prompted reattendance included abdominal, back, chest or shoulder pain, dyspepsia and change in bowel habit. 11% (32/296) of patients had previously been diagnosed with another cancer. The length of time a symptom had been present for was measured from first presentation to time of diagnosis. All prediagnosis serum measurements of bilirubin (345 tests), glucose (188 tests) and haemoglobin (335 tests) were also obtained for this cohort. A rising trend in glucose and bilirubin nearest to the time of diagnosis was observed (figure 2).

Very few biologically plausible symptoms were reported more than 1 year prior to diagnosis (table 1 and figure 1A, B). The limit of the study period was therefore set at 2 years, for the subsequent case–control study.

### Case–control study

In total, 2773 patients with PDAC, 848 patients with BTC and 15 395 controls were included in this study (table 2). In the year prior to diagnosis, patients with PDAC visited their GP on a median of 18 (IQR 11–27) occasions and patients with BTC visited their GP on a median of 22 (IQR 12–22) occasions. This is compared with control patients who visited their GP on a median of 14 occasions (IQR 8–21). In PDAC a median of three of these visits were with alarm symptoms and a quarter visited their GP on more than four occasions with alarm symptoms in the year prior to diagnosis.

**Table 2:** Patient characteristics

	Biliary tract cancer	Pancreatic cancer	Comparator group
	n=848 (%)	n=2773 (%)	n=15 395 (%)
Males (%)	390 (46)	1328 (48)	7233 (47)
Mean age (±SD)	71 (12)	72 (12)	71 (11)
Mean BMI (±SD)	27 (5)	27 (5)	27 (5)
Smoking status (%)			
Never	446 (53)	1345 (49)	8610 (56)
Ex	181 (21)	586 (21)	3635 (24)
Current	203 (24)	760 (27)	3090 (20)
Missing	18 (2)	82 (3)	60 (1)
Townsend score (%)			
(Most affluent) 1	194 (23)	739 (27)	4176 (27)
2	197 (23)	685 (25)	3611 (24)
3	168 (20)	542 (20)	3176 (21)
4	140 (17)	457 (17)	2536 (17)
(Most deprived) 5	128 (15)	290 (11)	1542 (10)
Missing	21 (2)	60 (2)	354 (2)

Body mass index (BMI)=weight (kg)/(height(m))^2^.

BMI<18.5=underweight, BMI 18.5–25=normal, BMI 25–30=overweight, BMI >30=obese.

In the 2 years prior to diagnosis, alarm symptoms were more common in patients with PDAC or BTC compared with controls (table 3). For example, 43.9% of patients with PDAC (OR=6.38 (95% CI 5.81 to 7.02)) and 37.3% of patients with BTC (OR=4.68 (95% CI 4.01 to 5.47)) consulted their GP with abdominal pain compared with just 11.3% of the control population. The incidence of dysphagia and pain other than abdominal or back was similar across all patient groups.

**Table 3:** Results from multivariable logistic regression models outlining the frequency and adjusted ORs of symptoms and signs of pancreatic cancer and BTC presenting to primary care in the 2 years prior to diagnosis compared with a control population without a cancer diagnosis

	Biliary tract cancer	Pancreatic cancer	Controls	Pancreatic cancer vs control			Biliary tract cancer vs control			Pancreatic cancer vs biliary tract cancer		
Symptom (%)	n=829	n=2790	n=17 192	OR*	95% CI	p Value	OR*	95% CI	p Value	OR*	95% CI	p Value
Weight loss	46 (5.5)	294 (10.5)	302 (1.8)	6.6	5.54 to 7.86	<0.001	3.17	2.32 to 4.34	<0.001	2.00	1.49 to 2.68	<0.001
Abdominal pain	309 (37.3)	1225 (43.9)	1946 (11.3)	6.38	5.81 to 7.02	<0.001	4.68	4.01 to 5.47	<0.001	1.35	1.15 to 159	<0.001
Nausea and vomiting	126 (15.2)	463 (16.6)	978 (5.7)	3.43	3.00 to 3.91	<0.001	2.99	2.44 to 3.66	<0.001	1.12	0.89 to 1.39	0.33
Bloating	27 (3.3)	113 (4.1)	229 (1.3)	3.1	2.48 to 3.89	<0.001	2.35	1.57 to 3.53	<0.001	1.25	0.82 to 1.89	0.298
Dyspepsia	118 (14.2)	559 (20)	1597 (9.3)	2.56	2.30 to 2.85	<0.001	1.7	1.40 to 2.08	<0.001	1.51	1.23 to 1.86	<0.001
New onset diabetes	48 (5.8)	380 (13.6)	1037 (6)	2.46	2.16 to 2.80	<0.001	0.92	0.68 to 1.23	0.559	2.59	1.91 to 3.52	<0.001
Change in bowel habit	194 (23.4)	764 (27.4)	2557 (14.9)	2.17	1.98 to 2.39	<0.001	1.77	1.51 to 2.09	<0.001	1.23	1.04 to 1.46	0.014
Pruritus	91 (11)	147 (5.3)	526 (3.1)	1.73	1.43 to 2.10	<0.001	3.75	2.96 to 4.74	<0.001	0.44	0.34 to 0.59	<0.001
Lethargy	71 (8.6)	293 (10.5)	1308 (7.6)	1.42	1.25 to 1.61	<0.001	1.08	0.83 to 1.40	0.57	1.28	0.97 to 1.68	0.086
Back pain	111 (13.4)	446 (16)	2111 (12.3)	1.33	1.18 to 1.49	<0.001	1.01	0.82 to 1.26	0.913	1.28	1.01 to 1.61	0.037
Dysphagia	10 (1.2)	51 (1.8)	254 (1.5)	1.21	0.90 to 1.64	0.206	0.81	0.43 to 1.51	0.498	1.51	0.76 to 3.03	0.241
Non-cardiac chest pain	114 (13.8)	335 (12)	2055 (12)	1.02	0.91 to 1.16	0.699	1.19	0.97 to 1.46	0.092	0.86	0.70 to 1.07	0.175
Shoulder pain	47 (5.7)	137 (4.9)	1052 (6.1)	0.78	0.65 to 0.93	0.006	0.86	0.64 to 1.14	0.294	0.87	0.63 to 1.18	0.367
Jaundice *	358 (43.2)	860 (30.8)	36 (0.2)	246.00	172 to 351	<0.001	445	302 to 658	<0.001	0.57	0.48 to 0.66	<0.001

Models were adjusted for age, gender, time period and social deprivation (each symptom was assessed independently).

*Only 0.2% of controls were recorded with jaundice. However, among patients with PDAC and BCT, jaundice was highly prevalent occurring in 43.2% of patients with BTC and 30.8% of patients with PDAC which lead to extremely large OR estimates.

BTC, biliary tract cancer; PDAC, pancreatic ductal adenocarcinoma.

When comparing the symptom profiles of PDAC and BTC, some symptoms were only a feature of PDAC such as back pain, lethargy or new onset diabetes. However, other alarm symptoms did overlap between both cancers but generally were a more common feature of one cancer than the other. For example, abdominal pain (OR=1.35 (95% CI 1.15 to 1.59)) weight loss (OR=2.00 (95% CI 1.49 to 2.68)) and dyspepsia (OR=1.51 (95% CI 1.23 to 1.89)) were more frequently associated with PDAC and jaundice (OR=0.44 (95% CI 0.34 to 0.59)), and pruritus (OR=0.57 (95% CI 0.48 to 0.66)) was more frequently associated with BTC (table 3). Symptoms of unexplained weight loss were around twice as common in patients with PDAC compared with patients with BTC.

Mean liver biochemical tests including serum bilirubin, ALP and ALT closest to the date of diagnosis were substantially higher in patients with PDAC and BTC compared with controls (p<0.001; table 4). Mean serum bilirubin levels in BTC (26.1 µmol/L) and PDAC (20.7 µmol/L) were higher than in controls (10.2 µmol/L) but not at clinically detectable levels. The mean levels of bilirubin and ALP in patients with BTC were around double those of the control patients. With the exception of ALP, which was significantly higher in BTC compared with PDAC (p<0.001), there was no significant difference in routinely performed blood tests between the two cancer types (table 4).

**Table 4:** Liver function tests, haemoglobin levels and BMI in patients with pancreatic cancer and biliary tract cancer compared with a control population without a cancer diagnosis

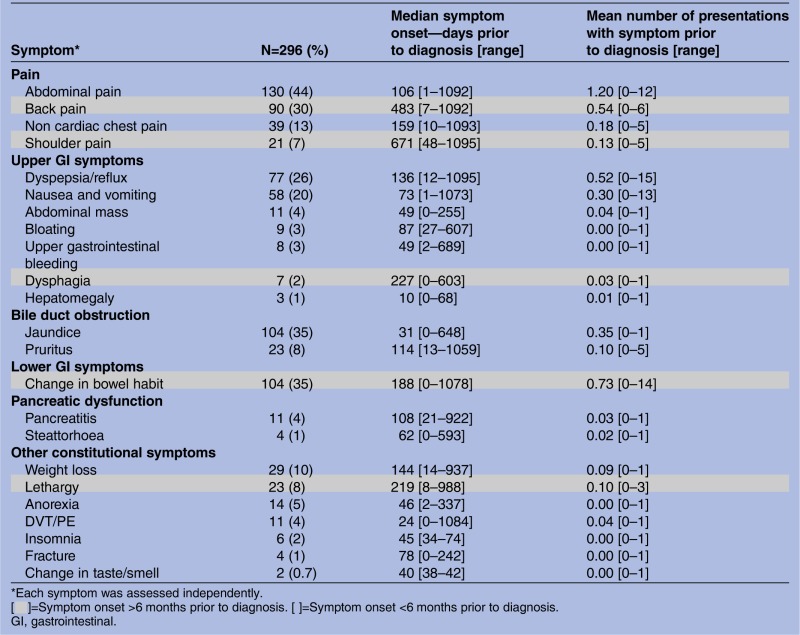

BMI was significantly lower in patients with PDAC compared with patients with BTC or control patients. However, adjustments for BMI and smoking status had no meaningful effect on any of the relationships reported.

## Discussion

This study further defines the early symptom profile of PDAC and for the first time defines early alarm symptoms that are associated with BTC in a primary care population. Prior to this study, very little was known about early alarm symptoms in BTC^23^ ^24^ and how they overlapped with PDAC, although the clinical presentation of the two cancers was recognised to be similar. Associated alarm symptoms in BTC and PDAC in this study by and large reflected the underlying pathology and correlated with disease progression (tables 1 and 3).

With the exception of jaundice, the individual ORs of many of the associated symptoms in PDAC and BTC were low. However, nearly all patients reported alarm symptoms and often attended their GP on several occasions in the year prior to diagnosis. To aid the earlier diagnosis of these cancers, this study would therefore support the development of CDSTs, which incorporate multiple early onset, alarm symptoms.

A recent validation study of an existing CDST for PDAC suggests it may over predict cancer risk in certain groups including older patients.^10^ This has the potential to cause unnecessary anxiety for patients and substantially increase workloads in hospital departments due to extra referrals for the investigation of patients with suspected cancer. Further refinement of existing tools to improve their diagnostic accuracy is therefore likely to be necessary.

Patients with PDAC or BTC in this study visited their GP more frequently in the 2 years prior to diagnosis. This reflected trends found in other primary care studies^7^ and large patient surveys (National Cancer Patient Experience Survey).^13^ A change in attendance behaviour should therefore be considered as an alarm feature for cancer, particularly if patients reattend with the same alarm symptom or a constellation of alarm symptoms.

Apart from one other retrospective secondary care study, the length of time symptoms are present in patients with PDAC has received little attention.^8^ By comparison, pruritus appeared to be reported earlier in this primary care cohort but other symptoms such as change in bowel habit and anorexia were present for a similar length of time. Previous studies measured symptom onset in accordance with the development of jaundice or abdominal pain rather than final diagnosis as in this study, which may account for some of the discrepancies observed.^8^

Identified early symptoms of PDAC (table 1) were similar to those identified in other primary and secondary care studies.^6–8^ However, dyspepsia and pruritus have not been identified as alarm symptoms for PDAC in primary care patients previously.^6^ ^7^ Dysphagia was identified in another primary care study as an independent predictor of PDAC in men,^6^ however it was not found to be a common early symptom in this study. Significant overlap occurred in the early symptoms of PDAC and BTC and may account for why these tumours are often difficult to differentiate preoperatively. However, even in these two malignant conditions that are recognised to present similarly, certain symptoms such as back pain, lethargy and new onset diabetes were identified as unique features of PDAC. Hence, when designing future CDSTs, symptom overlap and the inclusion of unique symptoms should be a design consideration.

The frequency of alarm symptoms in this study was similar to other primary care studies^6^ ^7^ but lower than those reported in retrospective secondary care studies.^6–8^ This trend has been reported before^7^ and may reflect that there are some symptoms for which patients do not seek medical advice. For example, anorexia and change in taste were seen with much greater frequency in secondary care than primary care studies. If CDSTs are to be used by patients directly in the future, further modification of these tools in line with their pre-presentation symptom profile will be necessary.

Commonly performed blood tests, in particular liver function tests (bilirubin, ALT, ALP), often became abnormal prior to diagnosis. These tests are frequently performed by GPs as part of drug monitoring and routine health checks and therefore could be included in future algorithms.

Further guidance on the best methods of managing patients with suspected PDAC or BTC in primary care is also urgently needed. Current UK guidance on referring patients with suspected PDAC or BTC from primary care is incorporated within guidance for all upper gastrointestinal cancers.^14^ These recommendations focus on the exclusion of oesophagogastric cancer through urgent gastroscopy, which is often normal in patients with PDAC or BTC. The lack of specific information about recognising PDAC or BTC and optimal methods for further investigation has the potential to cause further delays in diagnosis. The inclusion of more information about alarm symptoms for PDAC and BTC, the use of CDST in routine practice and thresholds for investigation would be particularly valuable to GPs. Ultimately alignment of these tools to rapid assessment pathways could prevent outpatients and diagnostic services becoming overwhelmed.

## Conclusions

Referrals for investigation of suspected PDAC or BTC from primary care is currently dependent on symptom recognition. Further definition of early alarm symptoms associated with these two cancers by this study will support GPs in identifying patients with suspected PDAC or BTC. The information will also inform the future modification of current symptom-based CDSTs. Widespread use of these tools in primary care is expected to lead to patients being diagnosed at an earlier stage when curative therapy is possible. Subsequent improvements in overall survival are expected.
